# 

**Published:** 1869-04

**Authors:** 


*Dr. L. Stuck's Rubber Plates.*—In the February number of the *Journal* we published an article, taken from the *Missouri Dental Journal*, which professed to describe Dr. Stuck's method of constructing artificial dentures on vulcanized rubber, and at the same time condemned it as being one of the humbugs of the age.

During the latter part of the past session of the Baltimore College of Dental Surgery, Dr. Stuck visited Baltimore and explained his method of manipulating rubber in such a satisfactory manner, that we were induced to give it a trial in our office laboratory.

The experience we have had with this process since Dr. Stuck's visit, enables us to speak of it in the highest terms, and recommend it to our readers.

Besides giving a uniform thickness to that portion of the plate covering the hard palate, we obtain by Dr. Stuck's process an accurate adaptation to the mouth which enables us to give perfect satisfaction in many difficult cases. The fact of the plate or set leaving the flask with a beautiful and durable polish on every part except the mere rim, thereby saving great labor as well as time, is enough of itself to commend Dr. Stuck's process.

The uniform thickness of the plate, and the hardening of the rubber between pure tin surfaces, gives an elasticity to the set which is both serviceable and comfortable in the mouth.

We understand that at the time the agent referred to by the *Missouri Dental Journal*, visited St. Louis, the process was not perfected, and, also, that this agent did not fairly represent the inventor; promising more than he was authorizod to do. This being the case, the writer of the article referred to, may have been justified in speaking lightly of this process, although at the same time he expresses the hope that some one will take the hint suggested by this method of Dr. Stuck's and get up something more practical.

---

*Dr. McClelland's Rose-Pearl Base.*—For nearly two years past we have from time to time heard of Rose-Pearl, and it has been a matter of surprise that this material has not been brought directly before the profession.

At the Niagara meeting of the American Dental Association, some specimens of this work were on exhibition, and from the appearance they present-

### TO READERS AND CORRESPONDENTS.

Communications intended for publication, and exchange journals and papers, should be addressed to the EDITORS OF THE AMERICAN JOURNAL OF DENTAL SCIENCE, Baltimore, Md. All letters relating to the business of the journal, or containing cash remittances, to be directed to Messrs. SNOWDEN & COWMAN. Articles for publication should be received by the editors at least three weeks previous to the first day of the month on which the number in which it is designed for them to appear, is issued.

☞ The American Journal of Dental Science will contain fifty pages of reading matter in each number, making a volume of not less than *Six Hundred* pages;

EXCLUSIVE OF ADVERTISEMENTS.

